# Comprehensive HIV knowledge and associated factors among reproductive-age women: analysis of the Gambia Demographic and Health Survey 2019/2020

**DOI:** 10.1186/s12961-024-01128-4

**Published:** 2024-04-08

**Authors:** Menen Tsegaw, Bezawit Mulat, Kegnie Shitu, Amadou Barrow

**Affiliations:** 1 Department of Health Education and Behavioral Sciences, Institute of Public Health, College of Medicine and Health Sciences, University of Gondar, Gondar, Ethiopia; 2 Department of Human Physiology, School of Medicine, College of Medicine and Health Sciences, University of Gondar, Gondar, Ethiopia; 3 Department of Public Health, College of Medicine and Health Sciences, Ambo University, Ambo, Ethiopia; 4 Department of Public and Environmental Health, School of Medicine and Allied Health Sciences, University of the Gambia, Kanifing, The Gambia

**Keywords:** HIV/AIDS, Comprehensive knowledge, Gambia

## Abstract

**Introduction:**

Globally, there are 37.7 million people living with human immunodeficiency virus (HIV). So far, there is no study done in Gambia which assessed comprehensive HIV knowledge and its associated factors. Therefore, this study aims to assess comprehensive HIV knowledge and its associated factors among reproductive-age women in Gambia.

**Objective:**

To assess the prevalence of comprehensive HIV knowledge and its associated factors among reproductive-age women in Gambia.

**Methods:**

The study used the Gambian Demographic and Health Survey, which was conducted from 21 November 2019 to 30 March 2020 in Gambia. The survey employed a stratified two-stage cluster sampling technique to recruit study participants. Logistic regression analysis was used to identify factors associated with HIV comprehensive knowledge. Statistical significance was declared at a *P* value of less than 0.05 with a 95% confidence interval (CI).

**Results:**

The overall prevalence of comprehensive HIV knowledge was 27.1% (25.1–36.2%). Older age [adjusted odds ratio (AOR) of 1.20 (95% CI 1.16–1.26)], using contraceptive [AOR of 1.15 (95% CI 1.01–1.31)], higher education [AOR of 4.73 (95% CI 3.86–5.81)], rich wealth quintile [AOR of 1.61 (95% CI 1.37–1.87)], media exposure [AOR of 1.76 (95% CI 1.39–2.23)], ever tested for HIV [AOR of 1.55 (95% CI 1.42–1.74)], visited health facility within the last 12 months [AOR of 1.26 (95% CI 1.12–1.41)] and decision-making autonomy [AOR of 1.42 (95% CI 1.27–1.60)] were positively associated with comprehensive HIV knowledge. However, being married [AOR of 0.72 (95% CI 0.62–0.82)] was negatively associated with comprehensive HIV knowledge.

**Conclusions:**

The prevalence of comprehensive HIV knowledge was low in Gambia. Educational interventions that focused mainly on awareness creation about HIV/AIDS should be designed especially for married women and lower socio-economic status. An effort has to be made to address those disparities at the national level.

## Introduction

Comprehensive knowledge (CK) about human immunodeficiency virus (HIV) includes an understanding of HIV transmission routes and the ability to reject local misconceptions about human immunodeficiency virus/acquired immune deficiency syndrome (HIV/AIDS) transmission [1, 2]. Globally, there are 37.7 million people living with human immunodeficiency virus (HIV), and 680 000 people died from HIV-related causes in 2021 [3, 4]. In 2022, globally 1.3 million people acquired HIV and 630 000 people died from HIV-related causes [5]. HIV is a major global public health problem with a highest burden in sub-Saharan Africa accounting 70% of the global burden of infections [6, 7]. In 2014, the Joint United Nations Program on HIV/AIDS (UNAIDS) and partners set the 90–90–90 target for the year 2020: diagnose 90% of all people living with HIV (PLHIV), treat 90% of people who know their status and suppress the virus in 90% of people on treatment [8]. Among all PLHIV worldwide, 79% knew their HIV status; of these, 78% were accessing treatment, and 86% of people accessing treatment had suppressed viral loads in 2018 [9]. Comprehensive HIV knowledge is very important for sexual health and behaviour [10]. Many studies conducted on comprehensive knowledge have found that age, educational level, ever tested for HIV, media exposure and wealth are the independent predictors of comprehensive HIV knowledge [1, 11–14].

To achieve the 90–90–90 plan of United Nations (UN) ending AIDS, the Catch Up Plan (2018–2020) was launched in Gambia, which mainly targeted scaling up voluntary counselling and testing services [15]. According to the Gambia Demographic and Health Survey (GDHS) 2019–2020, the prevalence of comprehensive HIV knowledge was 27% among reproductive-age women [16]. Lack of comprehensive knowledge about HIV/AIDS is a major public health problem, which contributed for the increased number of new HIV infections and burdens for reproductive-age women (15–49 years) [17–21].

There is scanty information on the factors affecting comprehensive HIV knowledge among reproductive-age women in Gambia. Therefore, the current study aims to identify factors associated with comprehensive HIV knowledge among reproductive-age women in Gambia.

## Methods

### Study design and setting

This study used secondary data extracted from the Gambia Demographic and Health Survey 2019–2020. A total of 88 participants (60 women and 28 men) were trained on the paper questionnaires. Each team consisted of six members, typically with the following composition: one supervisor, three female interviewers, one male interviewer and one biomarker technician. In the interviewed households, 12,481 women aged 15–49 years were identified for individual interviews; the interviews were completed with 11,865 women, yielding a response rate of 95%. The survey employed a stratified two-stage cluster sampling. In the first stage, enumeration areas (EAs) were selected with a probability proportional to their size within each sampling stratum. In the second stage, the households were systematically sampled. A total of 11,865 weighted samples of reproductive-age women were included in the study. Gambia is located on the West African coast between latitude 13° and 14° north and 13° and 17° west. It is bordered on the north, south and east by the Republic of Senegal and on the west by the Atlantic Ocean.

## Variables of the study

### Dependent variable

Comprehensive HIV knowledge was the outcome variables of this study which was defined as the correct knowledge of two mechanisms to prevent HIV and rejection of two misconceptions about HIV. To assess the comprehensive HIV and AIDS knowledge of reproductive-age women, every woman was asked whether or not she correctly answered the following five questions. A woman was considered as having comprehensive HIV knowledge if she knows that: (1) Can using condoms prevent HIV transmission? (2) Can HIV be prevented by limiting sex to one faithful uninfected partner? (3) Can a healthy-looking person have HIV? A woman was also considered as having comprehensive HIV knowledge if she rejects common misconceptions such as: (1) Can a person get HIV from mosquito bites? (2) Can a person get HIV by sharing food with someone infected? Accordingly, comprehensive knowledge of HIV/AIDS coded as non-knowledgeable women = 0 and knowledgeable women = 1 [11, 14, 22].

### Independent variables

Age of participants, sex of household head, marital status, educational status, wealth index (poor, middle or rich), media exposure (yes or no), type of place of residence (rural or urban), religion, working status (yes or no), history of HIV testing (yes or no),contraceptive use (yes or no), internet use (yes or no) and visited health facility within the last 12 months (yes or no). Decision-making autonomy (yes or no) were independent variables of the study. We have extracted those variables by reviewing different relevant literature [10, 17, 21–24].

### Data analysis

Data cleaning was performed to check any missing values. Recoding, labelling and statistical analysis were performed according using STATA version 14.0. The women’s individual sample weightings were used in the estimation to provide nationally representative data. Descriptive measures, such as frequency mean and percentages, were computed. Bi-variable logistic regression was used to select candidate variables for multi-variable logistic regression. In the bi-variable logistic regression, a *P* value of less than 0.2 was used as a cut of point to select variables for the multi-variable analysis entry. Multi-variable logistic regression was used to identify independent predictors of comprehensive HIV knowledge among reproductive-age women in Gambia by controlling confounders. A 95% confidence interval (CI) and *P* value < 0.05 were used to determine the statistical significance. Multi-collinearity was checked and there was no the problem of multi-collinearity. All variables were entered at the same time and multi-variable logistic regression was conducted accordingly.

## Results

The mean ages of the participants were 28.21 (± 9.33) years with a range of 15–49 years. Among the participants, 44.7% were from Brikama region, 34.7% had no education, 73.7% were resided in urban area, 34.8% were from a poor household, 42.1% had tested for HIV and 67.1% has visited health facility with in the last 12 months (Table 1).

**Table 1:** Socio-demographic characteristics and its association with comprehensive HIV knowledge among reproductive-age women in Gambia (*n* = 11 865)

Variables	Comprehensive HIV knowledge		Total	Percent	*P* value
	Yes	No			
Age (years)					
15–19	503	2130	2633	22.2	< 0.001
20–24	576	1605	2181	18.4	
25–29	609	1639	2248	19.0	
30–34	532	1087	1619	13.7	
35–39	474	964	1438	12.0	
40–44	277	751	1028	8.7	
45–49	239	479	718	6.0	
Marital status					
Single	1008	2697	3705	31.2	0.016
Married	2202	5958	8160	68.8	
Current contraceptive use					
Yes	540	1052	1592	13.4	< 0.001
No	2670	7603	10,273	86.6	
Region					
Banjul	59	104	163	1.4	< 0.001
Kaniking	849	1740	2589	21.8	
Brikama	1529	3770	5299	44.7	
Mansakonko	84	347	431	3.6	
Kerewan	385	744	1129	9.5	
Kuntaur	80	442	522	4.4	
Janjanbureh	122	473	595	5.0	
Basse	102	1035	1137	9.6	
Women’s educational level					
No education	728	3391	4119	34.7	< 0.001
Primary	363	1491	1854	15.6	
Secondary	1623	3398	5021	42.3	
Higher	496	375	871	7.4	
Current working status					
Yes	1774	4215	5989	50.5	< 0.001
No	1436	440	5876	49.5	
Religion					
Christian	141	281	422	3.5	< 0.001
Islam	3069	8374	11,443	96.5	
Residence					
Urban	2599	6148	8747	73.7	< 0.001
Rural	611	2507	3118	26.3	
Wealth index					
Poor	746	3387	4133	34.8	< 0.001
Middle	568	1724	2292	19.3	
Rich	1896	3544	5440	45.9	
Media exposure					
Yes	3145	8008	11,153	94.0	< 0.001
No	65	647	712	6.0	
Sex of household head					
Male	2329	6899	9228	77.8	< 0.001
Female	881	1756	2637	22.2	
Ever tested for HIV					
Yes	1691	3310	5000	42.1	< 0.001
No	1519	5345	6864	57.9	
Use of internet					
No	2488	5295	7783	65.6	< 0.001
Yes	722	3360	4082	34.4	
Visited health facility in the last 12 months					
No	2298	5667	7965	67.1	< 0.001
Yes	912	2988	3900	32.9	
Decision-making autonomy					
Yes	631	1551	2182	27.0	< 0.001
No	1232	4669	5901	73.0	

### Magnitude of comprehensive HIV knowledge

**Fig. 1:**
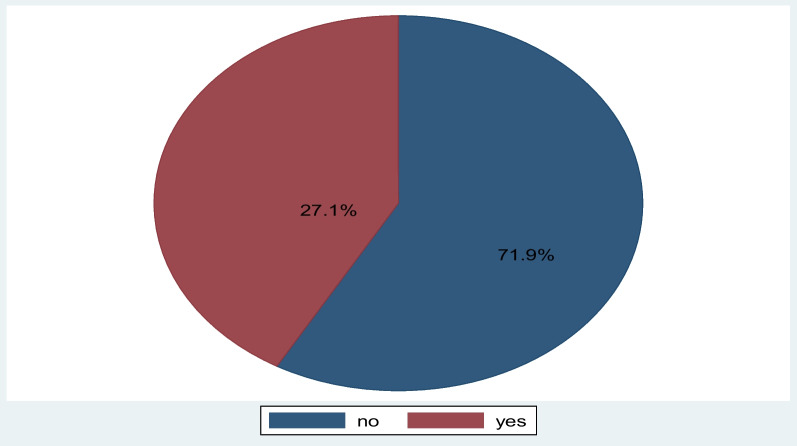
The prevalence of comprehensive HIV knowledge among reproductive-age women in Gambia (*n* = 11 865)

The overall prevalence of comprehensive HIV knowledge was 27.1% (25.1–36.2%) (Fig. 1).

### Factors associated with HIV comprehensive knowledge

In the bi-variable logistic regression age of the women, religion, region, educational level, working status, residence, wealth index, media exposure, marital status, sex of household head, current contraceptive use, visited a health facility, ever tested for HIV, decision-making autonomy and internet use were independently fitted to the outcome variable. All those variables were candidates for multi-variable logistic regression at a *P* value less than 0.2 and were statistically significant.

In multi-variable logistic regression, age of the women, region, educational level, wealth index, media exposure, marital status, contraceptive use, ever tested for HIV, internet use and visited health facility within the last 12 months were significantly associated with the comprehensive HIV knowledge among reproductive-age women in Gambia.

Women whose age found above the age group of 15–19 years were 1.21 times more likely to have higher odds of comprehensive HIV knowledge than those found within the age group of 15–19 years [adjusted odds ratio (AOR) of 1.20 (95% CI 1.16–1.26)]. The odds of comprehensive HIV knowledge was reduced by 28% among married women as compared with single women [AOR of 0.72 (95% CI 0.62–0.82)].

The odds of having comprehensive knowledge on HIV were 1.88 [AOR of 1.88 (95% CI 1.53–2.32)] and 1.30 [AOR of 1.30 [95% CI 1.04–1.64)] times higher among women residing in Kerewan and Janjanbureh respectively as compared with those from Banjul. However, residing in Basse reduces the odds of having comprehensive HIV knowledge by 60% [AOR of 0.40 (95% CI 0.32–0.51)]. The odds of having comprehensive knowledge about HIV were 1.15 times higher among contraceptive users as compared with their counterparts [AOR of 1.15 (95% CI 1.01–1.31)].

Women with secondary and higher educational level were 2.17 (AOR of 1.91 (95% CI 1.91–1.49)] and 4.73 [AOR of 4.73 (95% CI 3.86–5.81)] times higher as compared with those with no education respectively. Comprehensive knowledge on HIV were 1.29 (AOR of 1.29 (95% CI 1.11–2.45)] and 1.61 [AOR of 1.61 (95% CI 1.37–1.87)] times higher among women from households with middle and rich wealth quintiles as compared with those women from households with poor wealth quintiles. The likelihood of having good HIV comprehensive knowledge were 1.76 times higher among women who were exposed to media as compared with those who were not exposed [AOR of 1.76 (95% CI 1.39–2.23)].

The likelihood of having comprehensive HIV knowledge was 1.55 [AOR  of 1.56 (95% CI 1.42–1.74)] times higher among those women who had ever tested for HIV as compared with those who had not tested. Comprehensive HIV knowledge was 1.43 times higher among women who used internet as compared with those who did not use. The likelihood of having comprehensive HIV knowledge were 1.26 [AOR of 1.26 (95% CI 1.12–1.41)] times higher among women who had visited health facility within the last 12 months as compared with their counterparts. The odds of comprehensive HIV knowledge was 1.42 [AOR of 1.42 (95% CI 1.27–1.60)] (Table 2).

**Table 2:** Factors associated with HIV comprehensive knowledge among reproductive-age women in Gambia (*n* = 11 865)

Variables	Crude odds ratio (COR) (95% CI)	*P* value	AOR (95% CI)	*P* value
Age of the mother (years)	1.12 (1.09–1.14)	< 0.001	1.20 (1.16–1.26)*	< 0.001
Region				
Banjul(ref)	1		1	
Kaniking	0.89 (0.76–1.06)	0.194	0.89 (0.74–1.06)	0.187
Brikama	0.79 (0.68–0.92)	0.003	0.89 (0.75–1.05)	0.170
Mansakonko	0.47 (0.39–0.56)	< 0.001	0.90 (0.71–1.14)	0.396
Kerewan	0.74 (0.63–0.88)	0.001	1.88 (1.53–2.32)*	< 0.001
Kuntaur	0.28 (0.24–0.34)	< 0.001	0.92 (0.72–1.18)	0.509
Janjanbureh	0.47 (0.39–0.56)	< 0.001	1.30 (1.04–1.64)*	0.023
Basse	0.17 (0.14–0.21)	< 0.001	0.40 (0.32–0.51)*	< 0.001
Women’s educational level				
No education	1		1	
Primary	1.10 (0.97–1.25)	0.133	1.08 (0.93–1.25)	0.313
Secondary	2.57 (2.34–2.82)	< 0.001	2.17 (1.91–2.45)*	< 0.001
Higher	9.75 (8.08–11.76)	< 0.001	4.73 (3.86–5.81)*	< 0.001
Respondent currently working				
No	1		1	
Yes	1.19 (1.09–1.28)	< 0.001	0.97 (0.88–1.07)	0.541
Religion				
Christian/other	1		1	
Islam	0.51 (0.40–0.66)	< 0.001	1.03 (0.79–1.35)	0.818
Marital status				
Single	1		1	
Married	0.89 (0.81–0.98)	0.016	0.72 (0.62–0.82)*	< 0.001
Residence				
Urban	1		1	
Rural	0.49 (0.46–0.55)	< 0.001	0.95 (0.81–1.11)	0.516
Wealth index				
Poor	1		1	
Middle	1.49 (1.32–1.68)	< 0.001	1.29 (1.11–1.49)*	< 0.001
Rich	2.67 (2.42–2.93)	< 0.001	1.61 (1.37–1.87)*	< 0.001
Media exposure				
No	1		1	
Yes	3.72 (2.97–4.67)	< 0.001	1.76 (1.39–2.23)*	< 0.001
Sex of household head				
Male	1		1	
Female	1.57 (1.43–1.73)	< 0.001	0.95 (0.81–1.11)	0.516
Ever tested for HIV				
No	1		1	< 0.001
Yes	1.56 (1.45–1.69)	< 0.001	1.56 (1.41–1.73)*	
Visited health facility within the last 12 months				
No	1		1	
Yes	1.25 (1.15–1.37)	< 0.001	1.26 (1.12–1.41)*	< 0.001
Contraceptive use				
No	1		1	
Yes	1.47 (1.30–1.65)	< 0.001	1.15 (1.01–1.31)*	0.033
Use of internet				
No	1		1	
Yes	2.16 (1.97–2.37)	< 0.001	1.43 (1.28–1.59)*	< 0.001
Decision-making autonomy				
No	1		1	
Yes	1.54 (1.37–1.72)	< 0.001	1.42 (1.27–1.60)	< 0.001

*Statistically significant at *P* < 0.05

## Discussion

The aim of this study is to assess the prevalence of comprehensive HIV knowledge and its associated factors among reproductive-age women in Gambia. The prevalence of comprehensive HIV knowledge was 27.1% (25.1–36.2%). This finding is lower than the studies done in sub-Saharan Africa (SSA) [21]. This finding is higher than the study done in Myanmar [13]. The possible explanation for the discrepancy might be the differences in target population and study period. The possible reason for the discrepancy might be the difference in study period, number of countries involved in the study and type of study design. The study conducted in SSA involved many countries which may result in difference in prevalence of comprehensive HIV knowledge; this may be due to availability of different strategies, policies, interventions and initiatives implemented to enhance comprehensive HIV knowledge in those countries. The difference in study period may also result in difference in the level of comprehensive HIV knowledge due to the possibility of designing of various behaviour change intervention programs in different time periods across countries.

The age of the women was significantly associated with comprehensive HIV knowledge. Older-age women were more likely to have comprehensive HIV knowledge than younger ages. This finding is in line with the studies done in sub-Saharan Africa, Uganda, India and Nigeria [11, 21, 22, 24]. This might be due to the fact that older women are matured, and they may search information about their health. The other possible reason might be older women might feel free to visit a health facility to get information about their general health condition, and they might understand their susceptibility to diseases as well as the dysfunctional outcomes associated with it [25]. This implies that policy makers, health practitioners and health managers should consider age variation when they are designing strategies or interventions to enhance comprehensive HIV knowledge among reproductive-age women in Gambia.

Marital status was significantly associated with comprehensive HIV knowledge. Married women were less likely to have comprehensive HIV knowledge than single women. This result is consistent with the studies done in Ghana and Ethiopia [10, 23]. The possible reason might be that married women might believe that marriage is protective of HIV/AIDS and may not seek information about HIV transmission as well as prevention methods [22].

Local geographic area was significantly associated with comprehensive HIV knowledge. Women who reside in Kerewan and Janjanbureh regions of Gambia were more likely to have comprehensive HIV knowledge than those who reside in Banjul region. Contrary to this, women who reside in Basse were less likely to have comprehensive HIV knowledge as compared with Banjul. The possible reason might be that there may be spatial differences in access and availability of media or other information system about HIV. There may also be inter and intra-regional socio-cultural variation, which may either impede or facilitate HIV knowledge. The other possible reason might be that there may be educational level differences and differences in wealth quintile, which may in turn results in variation of comprehensive HIV knowledge across and within regions of Gambia. However, this needs further research of why this regional discrepancy occurs.

Current contraceptive use was significantly associated with comprehensive HIV knowledge. Those women who use contraceptives were more likely to have comprehensive HIV knowledge. This finding is in line with the study done in Ethiopia [17]. This might be due to the reason that women who used contraceptive methods might get information about HIV while they are going to the health facility. The other reason might be since condom use is one of the HIV and pregnancy preventive methods, a health professional might advise about the importance of using condoms and about HIV prevention, as well as transmission in general.

Educational level of the women was significantly associated with comprehensive HIV knowledge. Women with secondary and higher educational level are more likely to have higher comprehensive HIV knowledge than women with no education. This finding is in line with the studies done in Indonesia, Uganda and Bangladesh [18, 22, 26]. Educated women have decision-making power, which helps them to seek health information about HIV/AIDS and their health issues in general. They might have autonomy to decide on their health related aspects of life and could get access to health care services more easily. The other possible reason might be due to women attending secondary and higher education possibly getting HIV-related information either in the school curricula or in different extra-curricular activities, such as in different school clubs. Education may also help people to be curious about their health as well as to seek information for their health. Educated women may search different information sources, such as social media and internet websites of various organizations, who primarily target HIV, which may help them to clear out misperceptions there by helping to develop comprehensive knowledge [14, 27, 28]. This implies that health education should be provided for reproductive-age women about HIV. This finding suggests the need for an awareness creation campaign would be for reproductive-age women in Gambia to enhance their comprehensive knowledge about HIV/AIDS.

Wealth was significantly associated with comprehensive HIV knowledge. Women who were from middle and rich wealth quintiles were more likely to have comprehensive HIV knowledge than those from poor quintiles. This finding is in line with the studies conducted in Vietnam, Ethiopia and sub-Saharan Africa [17, 21, 29]. The possible reason might be women from higher wealth quintiles may have access to media and health information.

Media exposure was significantly associated with comprehensive HIV knowledge. Women who had exposed to media were more likely to have higher comprehensive HIV knowledge than their counterparts. This finding is in line with the studies done in Uganda, Ethiopia and Malawi [14, 22, 28]. Media has a great importance in creating awareness, enhancing knowledge and changing health behaviour of individuals as well as the community at large by delivering health information through the use of various channels [30]. This indicated the need for media involvement and coverage in transmitting health messages related with HIV.

If a woman was ever tested for HIV was significantly associated with comprehensive HIV knowledge. Women who had ever tested HIV were more likely to have higher comprehensive HIV knowledge as compared with their counterparts. This finding is in line with the studies done in Kenya, Uganda, Ethiopia and Vietnam [14, 22, 29, 31]. The reason might be women who had tested for HIV may get HIV counselling services by the health care provider about its transmission, as well as preventive methods.

Internet use was significantly associated with comprehensive HIV knowledge. Women who had used the internet were more likely to have higher comprehensive HIV knowledge than those who had not used the internet. This finding is in line with the studies done in Nigeria [32]. The internet has a substantial role in providing health information for various population groups including women [33]. This finding implies the need for disseminating health information related with HIV using different social media platforms or the internet.

Visiting a health facility in the last 12 months was significantly associated with comprehensive HIV knowledge. Women who had visited a health facility within the last 12 months were more likely to have comprehensive HIV knowledge than those who had not visited a health facility. The possible reason might be those who visited a health facility may get health education about different health and health-related issues during their waiting time. A women who visited a health facility for another purpose may get information on HIV at the health facility [1].

Decision-making autonomy was significantly associated with comprehensive HIV knowledge. Women who have autonomy to decide were more likely to have good comprehensive HIV knowledge as compared with their counterparts. This finding is in line with the study done in sub-Saharan countries and Pakistan [27, 34]. The possible reason might be because women who had decision-making capacity usually are more educated, have better information about HIV/AIDS, have better access to media and have better access to health care.

The findings of this research have an implication for public health research, practice and policy. The results of this study may help to design and strengthen public health policies to increase comprehensive HIV knowledge among reproductive-age women in Gambia.

## Strength

The use of sample weighting to overcome non-proportional sample allocation during the survey is one of the strengths of the study. The other strength of the current study is the use of a large sample size, which can help to increase the statistical power and validity of the study. Utilization of large sample size and national representativeness of Demographic and Health Surveys (DHS) data help the generalizability to the population of Gambia.

## Limitations

The study lacks causality. Therefore, a prospective study is needed to establish temporal association between the dependent variable and its covariates. There may be a chance of introducing recall bias. Due to the time gap between this study and data collection, those associations might not be the case now. The methodological limitation of this study is the use of multi-variable logistic regression analysis.

## Conclusions

The prevalence of comprehensive HIV knowledge was low in Gambia as compared with other study findings. Age, marital status, educational level, region, contraceptive use, wealth, media exposure, tested for HIV and visiting a health facility were significantly associated with comprehensive HIV knowledge. Health managers, policy makers and intended stakeholders working on this area should consider those factors while designing health intervention programs to enhance comprehensive HIV knowledge among reproductive-age women. Educational interventions that focus mainly on awareness creation about HIV/AIDS should be designed, especially for married women and women of lower socio-economic status. An effort has to be made to address those disparities at the national level.

## Data Availability

The datasets used and/or analysed during the current study are publicly available and can access at https://dhsprogram.com/data/dataset/Gambia_Standard-DHS_2019.cfm.

## Abbreviations

COR: Crude odds ratio
AOR: Adjusted odds ratio
CI: Confidence interval
GDHS: Gambia Demographic and Health Survey
